# Restoring T Cell Tolerance, Exploring the Potential of Histone Deacetylase Inhibitors for the Treatment of Juvenile Idiopathic Arthritis

**DOI:** 10.3389/fimmu.2019.00151

**Published:** 2019-02-07

**Authors:** Lotte Nijhuis, Janneke G. C. Peeters, Sebastiaan J. Vastert, Jorg van Loosdregt

**Affiliations:** Laboratory of Translational Immunology, Department of Pediatric Immunology & Rheumatology, University Medical Center Utrecht, University of Utrecht, Utrecht, Netherlands

**Keywords:** juvenile idiopathic arthritis (JIA), acetylation, HDAC inhibitor (histone deacetylase inhibitor), T cells, tolerance

## Abstract

Juvenile Idiopathic Arthritis (JIA) is characterized by a loss of immune tolerance. Here, the balance between the activity of effector T (Teff) cells and regulatory T (Treg) cells is disturbed resulting in chronic inflammation in the joints. Presently, therapeutic strategies are predominantly aimed at suppressing immune activation and pro-inflammatory effector mechanisms, ignoring the opportunity to also promote tolerance by boosting the regulatory side of the immune balance. Histone deacetylases (HDACs) can deacetylate both histone and non-histone proteins and have been demonstrated to modulate epigenetic regulation as well as cellular signaling in various cell types. Importantly, HDACs are potent regulators of both Teff cell and Treg cell function and can thus be regarded as attractive therapeutic targets in chronic inflammatory arthritis. HDAC inhibitors (HDACi) have proven therapeutic potential in the cancer field, and are presently being explored for their potential in the treatment of autoimmune diseases. Specific HDACi have already been demonstrated to reduce the secretion of pro-inflammatory cytokines by Teff cells, and promote Treg numbers and suppressive capacity *in vitro* and *in vivo*. In this review, we outline the role of the different classes of HDACs in both Teff cell and Treg cell function. Furthermore, we will review the effect of different HDACi on T cell tolerance and explore their potential as a therapeutic strategy for the treatment of oligoarticular and polyarticular JIA.

## Introduction

Juvenile Idiopathic Arthritis (JIA) is the most common rheumatic disease in children and an important cause of short- and long-term disability (1, 2). It includes several different entities and has an intriguing heterogeneity in disease course and outcome. Oligo-articular JIA (oJIA) has a relatively mild course, with lasting medication-free remission in approximately half of the children, while poly-articular JIA (pJIA) more often is non-remitting and can lead to severe disability (2, 3). The main pathophysiological concept of JIA is that the immunological balance is disturbed resulting in loss of immune tolerance (1). A unique subtype of JIA is systemic-onset JIA (sJIA), involving 10% of all JIA patients, and in contrast to oJIA and pJIA, sJIA is mainly characterized as an autoinflammatory disease instead of an autoimmune disease. In the pathogenesis of sJIA there is a key role for cells of the innate immune system including monocytes and neutrophils (4, 5). This is illustrated by the high incidence of macrophage activation syndrome (MAS) in patients with sJIA (6, 7). Although cells from the innate immune system play an essential role in the pathogenesis of oJIA and pJIA as well, it is generally thought that activation of autoreactive CD4^+^ T cells, leading to a T cell-driven immune response is a key manifestation in the pathogenesis of oJIA and pJIA. This subsequently results in recruitment of other immune cells, including innate immune cells and the production of several pro-inflammatory cytokines including tumor necrosis factor (TNF)α, interleukin (IL)-6, IL-17, and interferon (IFN) γ which collectively leads to joint inflammation. Innate immune cells, such as neutrophils and monocytes/macrophages to the site of inflammation (8). The pathogenic T cells present within the synovial compartment are predominantly Thelper (Th) 1 and Th17 cells (9, 10).

Regulatory T (Treg) cells are key players in maintaining immunological balance and tolerance (11, 12). The transcription factor forkhead box P3 (FOXP3) is crucial for Treg cell development and function and mutations in the *FOXP3* gene can result in severe dysregulation of the immune system due to a Treg cell deficiency (13, 14). Treg cell numbers and function have also been implicated in complex autoimmune diseases including rheumatoid arthritis (RA) and JIA, and in fact the first data on CD4^+^ Treg cells in human chronic arthritis comes from JIA patients (15, 16). Treg cells can be identified by the high expression of several markers, such as (but not limited to) FOXP3, CD25^high^, cytotoxic T lymphocyte associated protein (CTLA)-4 and low expression of CD127. Treg cells can adapt to local environment (tissues) and acquire additional characteristics in inflammatory conditions (12, 17). They seem to exert their regulatory or suppressive actions both cell-contact dependent and independent via the secretion of anti-inflammatory cytokines such as Transforming Growth Factor beta (TGF)β and IL-10 (18). In JIA, the balance between pro-inflammatory Teff cells and anti-inflammatory Treg cells can be associated with the course of the disease (16, 19–22). For instance, higher numbers of Treg and lower numbers of Teff cells (Th17 and Th1) at the site of inflammation have been correlated to a more favorable course and outcome in JIA (16, 20–22). These observations support the concept that treatment may be aimed to restore the immunological imbalance between effector mechanisms and regulatory mechanism in children with JIA.

Current treatment of JIA, consisting of intra-articular corticosteroids, disease modifying anti-rheumatic drugs (DMARDs) and biologicals, such as anti-TNFα, seem primarily directed at the effector side of the immunological imbalance (23–26). In the past two decades, biologicals are increasingly being used in JIA. They certainly have been a major- breakthrough in the treatment of JIA, but even today, a significant percentage of patients do not respond to therapy or only show partial response. Furthermore, after achieving clinical inactive disease on therapy, many patients suffer from relapse when treatment is discontinued (27, 28). Therefore, there is still a need for improved treatment strategies in chronic inflammatory diseases such as JIA. Restoring tolerance, either by; decreasing Teff cell function, increasing Treg cell function or preferentially both, might be a promising therapeutic strategy.

Histone deacetylases (HDACs) are a novel class of therapeutic targets that are being explored for the treatment of autoimmune disease. These enzymes can modulate epigenetic regulation and important cellular functions in many different cell types, including T cells by the deacetylation of both histone and non-histone proteins. In other diseases and research fields, mainly cancer research, HDAC inhibitors (HDACi) have already demonstrated therapeutic potential (29). Interestingly, in the context of autoimmune disease, HDAC inhibition proved to influence both the innate immune system and Teff cell and Treg cell function, potentially restoring immunological tolerance. We here provide an overview and focus on the role of the different types of HDACs in CD4^+^ Teff cells and Treg cells, and explore the potential of specific HDACi as a therapeutic strategy for the treatment of autoimmune diseases, in specific oJIA and pJIA.

## Histone Acetylation as Regulatory Mechanism of Immune Activation

**Figure 1:**
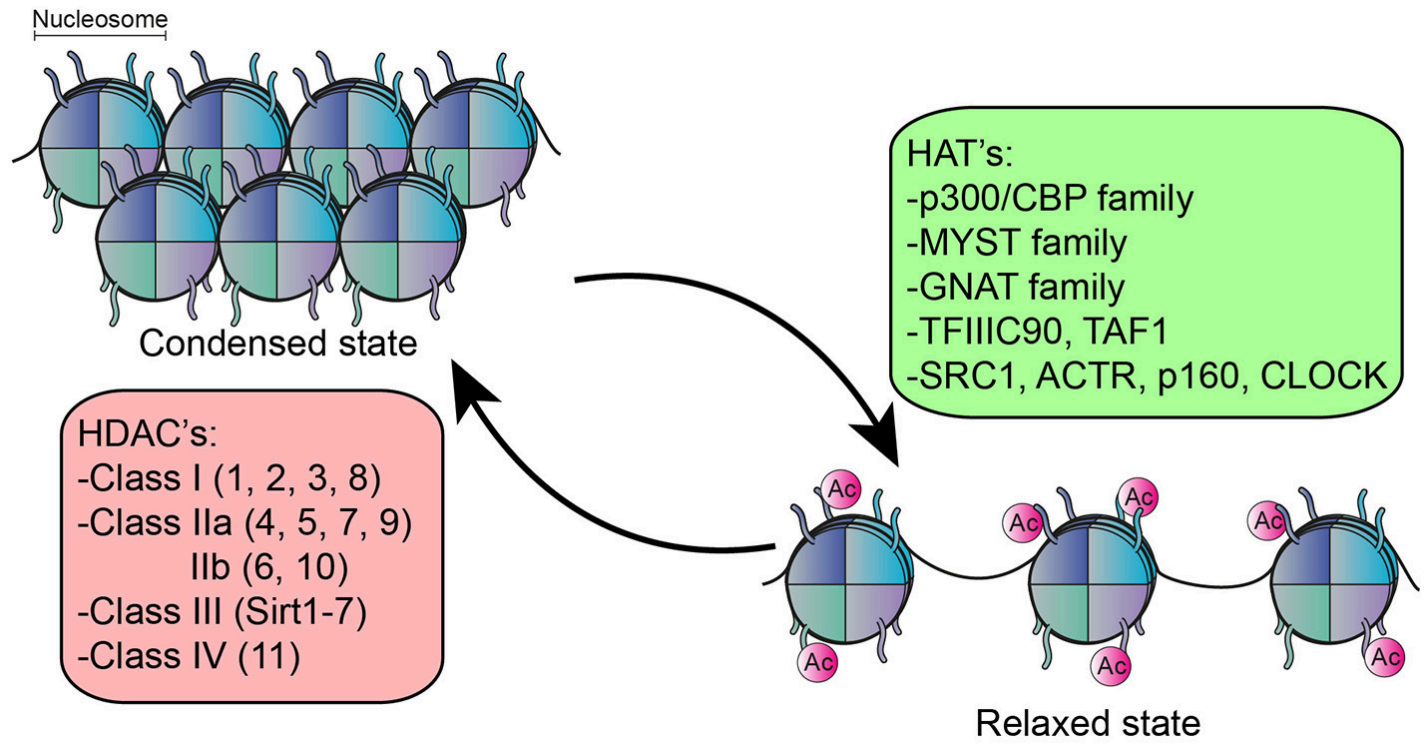
Function of HDACs an HATs. HATs acetylate the lysine residue on histones resulting in relaxation of chromatin structure, rendering the DNA more accessible for transcription factors. The reverse process, deacetylation by HDACs results in condensation of chromatin structure. 5 families of HATs have been described and 18 different HDACs, divided into 4 classes.

The function of many intracellular proteins, particularly transcription factors, and histones, can be altered by post-translational modifications. Here, one or more amino acids are covalently modified, often modulating subcellular localization, activation state, interaction with other proteins or protein turnover/degradation. Acetylation is one of the most prominent post-translational modifications. The majority of literature on acetylation is directed at its role in epigenetic regulation, which refers to changes in gene expression without altering the genetic code. In the nucleus, DNA is tightly wrapped around histones to form a nucleosome (30) which controls the accessibility of DNA binding sequence to their transcription factors (31). An important epigenetic mechanism that affects this accessibility is the post-translational modification of histones by acetylation (32), a process which is reciprocally regulated by lysine acetyl transferases (HATs) and lysine deacetylases (HDACs) (33–35) (Figure 1). In general, histone acetylation is associated with transcriptional activation by rendering the DNA more accessible to transcription factors (32, 36). The reverse process, deacetylation by HDACs, can therefore lead to condensation of chromatin structure and inhibition of gene transcription. However, deacetylation is also associated with activation of genes, and the inhibition of HDACs in fact results in both upregulation and downregulation of genes in equivalent percentages (37–41).

There are 18 different HDAC enzymes, which can be divided into 4 classes based upon homology to yeast HDACs and their function. Class I (HDAC1, 2, 3, and 8), Class II divided into class IIa (HDAC4, 5, 7, and 9) and IIb (HDAC6, 10) and class IV (HDAC11) are all Zinc-dependent and are considered classical HDACs. Class III consist of the Sirtuin family (Sirtuin 1–7) and are NAD^+^ dependent (42).

Histone modifications are widely associated with human disease, including malignancies and autoimmune disease such as RA, systemic lupus erythematosus (SLE) and JIA (35, 43–46). For instance, autoimmune disease associated single nucleotide polymorphisms (SNP's) are significantly enriched in regions with high acetylation of lysine 27 on histone 3 (H3K27) (46–48). Accordingly, increased regions of H3K27 acetylation in CD4^+^ T cells of JIA patients corresponded with increased expression of pro-inflammatory genes in these patients. Furthermore, in SLE, global acetylation of histone H3 and H4 in CD4^+^ T cells was reduced in patients compared to healthy controls and the degree of histone H3 acetylation negatively correlated with diseases activity (43). This demonstrates an important role for histone acetylation in autoimmune disease.

Next to histones, HDACs can also deacetylate non-histone proteins, hereby affecting their localization in the cell, stability and function (49, 50). For example, the activity of the transcription factors FOXP3 and Rar-related orphan receptor gamma (RORγt), key regulators of T cell function, is directly regulated by acetylation. Numerous studies have directly assessed the role of acetylation in modulating immune responses. Although there is a clear role for HDACs in the regulation of innate immune responses, reviewed by others (51, 52), we will focus on the role of the different HDACs in T cells and discuss their potential implications in oJIA and pJIA.

## HDAC Function in CD4^+^ T Cells

**Figure 2:**
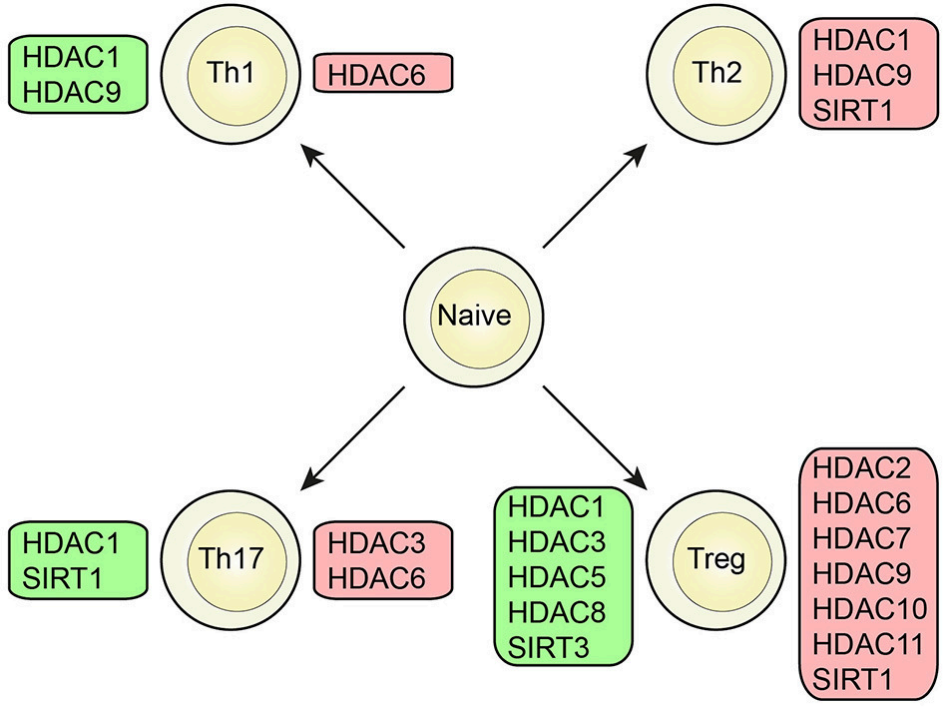
Role of HDAC members in different T cell subsets. Specific HDAC members which was demonstrated to either promote (green box) or inhibit (red box) function or differentiation of CD4^+^ T cells or the CD4^+^ T cell subsets; Th1, Th2, Th17, and Treg cells.

**Figure 3:**
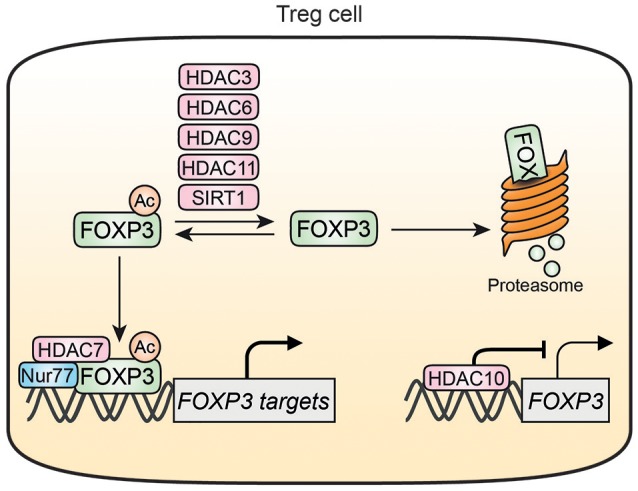
HDAC-mediated regulation of FOXP3 in Treg cells. HDAC3, HDAC6, HDAC9, HDAC11, and SIRT1 can deacetylate FOXP3 thereby increasing FOXP3 susceptibility for degradation. Acetylation of FOXP3 also promotes associations with DNA where it can form a transcriptional complex with HDAC7 and Nur77. HDAC10 can bind to and inhibit the FOXP3 promotor resulting in decreased FOXP3 expression. Nur77 is inhibited by HDAC7 and interacts with the HDAC7/FOXP3 transcriptional complex.

Several classes of HDACs were demonstrated to have an important function in CD4^+^T cell development and function (Figures 2, 3).

### Class I HDACs; HDAC 1, 2, 3, and 8

Several members of Class I HDACs are essential for T cell development and differentiation in mice. In mice, T cell-specific knock-out of *Hdac1* resulted in normal T cell numbers of both CD4^+^ and CD8^+^ T cells. However, these mice displayed enhanced Th2 responses, characterized by increased production of Th2 associated cytokines such as IL-4 and IL-5 combined with airway inflammation (53). In addition, *Hdac1* knock-out mice were resistant to the induction of experimental autoimmune encephalomyelitis (EAE), a multiple sclerosis animal model which is associated with Th1 and Th17 responses (54). These data suggest that HDAC1 can skew T cell responses by impairing Th2 function and potentiating Th1 and Th17 activity. Furthermore, HDAC1 can also modulate Treg function. In a mouse cardiac transplant model, the deletion of HDAC1 in FOXP3^+^ Treg cells resulted in an impaired function of these cells, combined with increased secretion of the pro-inflammatory cytokines IL-2, IL-17, IFNγ, and decreased cardiac allograft survival (55). This demonstrates that HDAC1 contributes to Treg cell suppressive capacity. While also T cell-specific deletion of HDAC2 did not affect T cell numbers, combined deletion of both HDAC1 and HDAC2 resulted in severe defects in mature T cell development, especially in the CD4^+^ T cell lineage. This indicates that HDAC1 and HDAC2 have overlapping functions in T cell development. In a combined *Hdac1/2* T cell knock-out mouse there was a decreased differentiation from double negative to double positive T cells (CD4^+^/CD8^+^) accompanied with a 5-fold decrease in thymocyte cellularity indicating a block in T cell development (56). *Hdac1/2* knock-out was proposed to result in defective propagation of T cell receptor (TCR) signaling. In a later stage of T cell development, the combined knock-out of *Hdac1/2* in CD4^+^ T cells specifically resulted in an increased CD8 surface expression by these CD4^+^ T cells and a decrease in peripheral T cell numbers (55). It was demonstrated that HDAC1 and HDAC2 maintain CD4 T cell integrity via repressing Runx-CBFß, a heterodimeric transcription factor required for the appropriate expression of CD4 and CD8. Furthermore, in T cells a dose dependent tumor suppressor function of HDAC1/2 was observed (56, 57). In Treg cells, the inhibition or deletion of HDAC2 specifically resulted in an increased Treg function, promoting cardiac allograft survival. This indicates that selective inhibition of HDAC2 in contrast to HDAC1 could serve a protective role against graft rejection (55). Combined targeting of both HDAC1 and 2 could result in severe defects in T cell development and therefore seems not suited for the treatment of JIA based upon data from mouse knock-out studies.

Comparable to the developmental defects observed in *Hdac1/2* knock-outs, HDAC3 also was shown to be essential for T cell development and maturation (58–60). Knock-out of *Hdac3* in mice resulted in a block in T cell development during positive selection in the thymus resulting in a strong reduction in CD4^+^ and CD8^+^ peripheral T cells. Within the CD4^+^ T cells present in the peripheral blood there was an increase in RORyt and IL-17 producing cells, indicating a skewing toward the Th17 phenotype (58). Furthermore, it was demonstrated that HDAC3 is an important mediator of the development and function of both induced and natural Treg cells. Interestingly, HDAC3 can directly associate with FOXP3, resulting in reduced IL-2 production by Treg cells. Moreover, mice with a FOXP3 cell-specific deletion of HDAC3 died within weeks from severe autoimmunity (61). For HDAC8, ongoing studies indicate that deletion of HDAC8 in Treg cells results in impaired Treg cell function (62). Together these data indicate that next to combined HDAC1/2 targeting, the selective inhibition of HDAC3 or HDAC8 could either result in T cell development defects or impaired Treg cell function and therefore does not seem to be of any therapeutic potential in a chronic autoimmune disease such as JIA.

### Class IIa HDACs; HDAC4, 5, 7, and 9

Most members from Class IIa HDACs are protective for the development of autoimmunity in mice. HDAC4 has been mainly assessed in the nervous system due to its high expression in brain and skeletal tissue and little is known about its function in T cells. Although HDAC4 is expressed in all T cell subsets, CD4^+^ T cells specific *Hdac4* knock-out mice display normal T cell numbers and function (63). A role for HDAC4 in CD4^+^ T cells was suggested based upon hypermethylation of the *HDAC4* region of the DNA of CD4^+^ T cells from RA patients. These data indicate that HDAC4 expression would be decreased in these patients, but gene expression or function of HDAC4 was not assessed in this study (64). Therefore, the exact role of HDAC4 in T cell and in autoimmune diseases is currently unknown.

Similarly, *Hdac5* knock-out mice display a normal CD4^+^ T cell development and function. In these mice however, Treg cell displayed impaired suppressive capacity (65). In addition, IFNγ production was decreased in CD8^+^ T cells, indicating that HDAC5 perhaps has a more profound role in CD8^+^ T cells and Treg cells compared to CD4^+^ Teff cells (65). In contrast to HDAC4 and HDAC5, HDAC7 is an important regulator of T cell development by regulating both positive and negative selection in the thymus (66–68). Knock down of *Hdac7* in mice T cells results in a defect in positive selection in the thymus with a decrease in T cell survival and TCR repertoire (66). The effect of HDAC7 on negative thymocyte selection was shown to be via inhibition of the expression of the transcription factor; Nur77 (69). Nur77 promotes thymocyte apoptosis during negative selection of autoreactive thymocytes (69) and the overexpression of Nur77 significantly decreases numbers of peripheral CD4^+^ and CD8^+^ T cells in mice (70). It was demonstrated that HDAC7 is recruited to the *Nur77* promotor via interaction with the transcription factor MEF2D resulting in decreased Nur77 expression. HDAC7 is exported out of the nucleus during T cell activation resulting in increased Nur77 expression. Blocking the nuclear export of HDAC7 in T cells in mice resulted in a block in negative selection in the thymus, promoted survival of auto-reactive T cells and was accompanied with the development of autoimmunity and a decreased life span in mice (67). The exact mechanism of inhibition of expression of Nur77 by HDAC7 remains unknown, but it is demonstrated to be dependent on its deacetylase activity (69). A profound role for HDAC7 in Treg function was also shown. Overexpression of Nur77 in mice T cells was associated with an increase in Treg cell percentages and cardiac allograft survival. It was demonstrated that Nur77 can interact with a HDAC7/FOXP3 transcriptional complex in Treg cells and that Nur77 overexpression resulted in increased expression of Treg associated genes, including Foxp3, Foxp1, TIP60 (70). Implicating, that Nur77 affects the balance between Teff and Treg cells, favoring Treg cell survival. Taken together, HDAC7 plays an essential role in negative T cell selection in the thymus as an inhibitor of Nur77. Both in Teff and Treg cells a protective role of Nur77 with respect to development of autoimmunity is suggested making Nur77 an interesting treatment target. However, the importance of HDAC7 in the positive selection of T cells in the thymus and TCR repertoire formation and therefore T cell development makes HDAC7 an undesirable target for the treatment of autoimmune diseases.

A role for HDAC9 has been implicated in several T cell subsets, including Treg, Th1, and Th2 cells both in mice and humans. HDAC9 expression was found to be increased in different subsets of CD4^+^ T cells of SLE patients and the autoimmune prone MLR/lpr mice. In addition, HDAC9 deficiency was associated with hyperacetylation of several lysine residues of histone H3 in mice (71). In the MLR/lpr mouse, knock-out of *Hdac9* resulted in a prolonged survival and decrease in autoimmune disease progression. This was determined by smaller lymph nodes and spleen and a decreased percentage of activated CD4^+^ T cells and double negative T cells. Furthermore, knock-out of *Hdac9* resulted in an inhibition of T cell activation *in vitro* and a decrease in Th1 and increase in Th2 cytokine production *in vivo* (71). Therefore, HDAC9 appears to promote skewing toward Th1 subsets. In addition, there is a differential expression of HDAC9 in different T cell subsets. HDAC9 is higher expressed in Treg cells compared to non-Treg cells and HDAC9 expression is markedly decreased in non-Treg T cells after stimulation in contrast to Treg cells (72). This could implicate an important role for HDAC9 in Treg function or development. Indeed, knock-out of HDAC9 in mice resulted in increased Treg numbers with enhanced suppressive function (73) and predisposition to iTreg development (74). This was confirmed by *in vitro* knock-down of *Hdac*9 in mice Treg cells which resulted in increased FOXP3 expression and increased suppressive function (73). Furthermore, the induction of colitis in mice was associated with increased HDAC9 expression, while the *Hdac9* knock-out mice were resistant to development of colitis (73). Taken together, HDAC9 showed to promote the development of autoimmune disease via its function in both Teff and Treg cells.

In summary class IIa HDACs in CD4^+^ T cells seem to be protective against the development of autoimmunity or essential for T cell development, with the exception of HDAC9. The inhibition of HDAC9 could therefore be of therapeutic interest in the context of autoimmune diseases such as JIA, however it's specific role in arthritis had not been investigated yet.

### Class IIb; HDAC6, 10

The two members of class IIb HDACs; HDAC6, and HDAC10 have both been assessed for their therapeutic potential as a new treatment of autoimmune disease. Importantly, in CD4^+^ specific *Hdac6* knock-out mice, CD4^+^ and CD8^+^ T-cell development and function was described to be normal (74–76). However, in these mice the population of IL-17 producing gamma delta (γδ) T cells was increased which was accompanied by a decreased expression of the transcription factor SOX4. This was confirmed *in vitro*, by treating a murine lymphoma cell line (EL4) with the two different HDAC6 inhibitors; tubacin, and tubastatin. HDAC6 inhibition in these cells resulted in a concentration-dependent increase of IL-17 expression (75). Since IL-17 production is associated with autoimmunity, these observations implicate a protective role for HDAC6 in the development of autoimmune disease via inhibition of Th17 cell differentiation. However, in Treg cells, it has been shown that HDAC6 is involved in deacetylation of FOXP3 and selective inhibition of HDAC6 enhanced the suppressive function of Treg cells (76–78). This was confirmed in an arthritis mouse model were HDAC6 inhibition resulted in decreased arthritis scores in mice (79). Furthermore, in human peripheral blood mononuclear cells (PBMCs) from RA patients, *in vitro* HDAC6 inhibition resulted in a decreased expression of the pro-inflammatory cytokines TNFα and IL-1β and increased the anti-inflammatory cytokine IL-10 (79). These observations indicate that HDAC6 can exert different effects in different T cell subsets, both pro-inflammatory and anti-inflammatory. Due to the observation that HDAC6 inhibition in Treg cells resulted in enhanced suppressive function and a decrease in arthritis development in a mouse model, there is great interest in the potential application of specific HDAC6i immune suppression therapy in for example transplant recipients. (62) However, more research is needed to determine the potential risk of HDAC6i by promoting autoimmune disease by increasing IL-17 production.

For HDAC10, it was reported very recently that it can bind to the *FOXP3* promotor and inhibit its transcriptional activity (80). Treg cells from HDAC10 knock-out mice showed increased expression of FOXP3 accompanied by increased Treg suppressive function. Furthermore, transfer of Treg cells from HDAC10 knock-out mice in a colitis mouse model resulted in a reduced induction of colitis compared to transfer of wild-type (WT) Treg cells (80).

Altogether, both HDAC6 and HDAC10, as class IIb HDACs, seem to exert negative effects on Treg cells and the inhibition of class IIb HDACs could therefore be beneficial in inducing Treg cell function. However, for HDAC6 there is a differential effect on different Th subsets and a pro-inflammatory effect has also been demonstrated via the induction of IL-17 production.

### Class IV; HDAC11

HDAC11, the only member of class IV HDACs is suggested to have an inhibitory role in maintaining immune tolerance. For Treg cells it was demonstrated in a human T cell line that HDAC11 can associate with and deacetylate FOXP3 (81). Knock-out of *Hdac11* in Treg cells resulted in an increased expression of FOXP3 and TGF-β, and an increased suppressive capacity *in vitro*. This was confirmed *in vivo* by an increased cardiac allograft survival in *Hdac11* knock-out mice (81). In mice, *in vitro* activation of Teff cells resulted in a downregulation of HDAC11 expression. In addition, *Hdac11* knock-out in CD4^+^ and CD8^+^ T cells resulted in an increased proliferation and pro-inflammatory cytokine production after activation indicating an inhibitory role for HDAC11 in Teff cell activation. Interestingly, the *Hdac11* knock-out CD4^+^ T cells were resistant to the *in vivo* induction of tolerance via the injection of a tolerogenic dose of ovalbumin (82). In summary, *Hdac*11 knock-out has a differential effect on the different T cell subsets with an increased Teff function and decreased tolerance induction after HDAC11 knock-out in all T cells. In contrast, Treg cell-specific knock-out of HDAC11 results in the opposite effect with an increased suppressive function of Treg cells and improved immune tolerance. The effect of HDAC11 inhibition *in vivo* therefore seems to depend on which cell type is affected most. To our knowledge, no specific inhibitor for HDAC11 is available yet and therefore the effect of HDAC11 inhibition *in vivo* remains incompletely understood.

### Class III; Sirtuins1-7

The most extensively studied member of the sirtuin family, SIRT1, is an important player in chronic inflammation. The exact function of SIRT1 is under debate and both a pro-inflammatory and an anti-inflammatory role of SIRT1 have been described in human disease. It was demonstrated that SIRT1 is upregulated in the synovial tissue and PBMC from patients with RA compared to patients with osteoarthritis. In this study, a pro-inflammatory role for SIRT1 was implied in monocytes with a reduction in lipopolysaccharide (LPS) induced TNFα production *in vitro* after inhibition of SIRT1, either by incubation with SIRT1 inhibitors or siRNA targeting (83). However, the opposing effect has also been demonstrated where a protective role for SIRT1 against inflammation and tissue destruction is suggested in chondrocytes and osteoblasts via the deacetylation and therefore inactivation of Nuclear factor-kappa B (NF-κB) (84, 85). Part of this controversy may be explained by differences in experimental setup, cell type and readout.

In mice T cells, the germline knock-out of *Sirt1* resulted in an increased T cell proliferation and expression of IL-2, IFNγ, and IL-5 compared to WT upon *in vitro* activation. In these mice, this decreased T cell tolerance resulted in increased development of EAE (86). In contrast to what is found in the germline knock-out mice, T cell development, cytokine expression and proliferation upon *in vitro* activation is normal in CD4^+^ T cell specific *Sirt1* knock-out mice (87). These observations indicate that the role of SIRT1 varies within different T cell subsets and different stages of T cell development. This is in line with the finding that basal sirtuin/SIRT1 levels differ between different T cell subsets. For instance, in mice, all sirtuin members are expressed in higher levels in Treg cells compared to Teff cells. Interestingly, upon *in vitro* activation via the T cell receptor there is a markedly increased expression of all sirtuin members in mouse Teff cells, but a downregulation of some sirtuins, including SIRT1, in Treg cells (76). Furthermore, SIRT1 plays an important role in the function of Treg cells via its effect on FOXP3. Both FOXP3 activity and stability is dependent on its acetylation status and it was demonstrated that SIRT1 associates with and deacetylates FOXP3, resulting in its degradation (55, 88–90). This was confirmed *in vivo* by Treg-specific *Sirt1* knock-out in mice where there was increased expression of FOXP3 (87). In addition, the increase in FOXP3 expression was accompanied by an increased suppressive capacity of Treg cells. Transfer of Treg cells from both *Cd4*^+^ and *Foxp*3^+^ specific knock-out mice into immune-deficient mice showed a more potent suppressive capacity compared to WT Treg cells (87). Moreover, in a colitis mouse model, the adoptive transfer of Teff cells isolated from CD4^+^ specific *Sirt1* knock-out mice resulted in a nearly 3-fold increase in iTreg formation compared with mice receiving WT Teff cells. This correlated with reduced weight loss and reduced development of colitis (91).

In other T cell subsets, treatment with an siRNA for *Sirt1*, resulted in increased of IL-9 production *in vitro* by both mouse and human CD4^+^ T cells which was accompanied with an increase in allergic airway inflammation in mice (89). In Th17 cells, *Sirt1* knock-out was demonstrated to inhibit Th17 differentiation via RORyt hyperacetylation and showed to be protective in an EAE mouse model (92). IL-9 producing Th9 cells are associated with a Th2 type response while Th17 cells are important pro-inflammatory players in autoimmune disease.

Altogether, the function of SIRT1 can exert varying effects on different T cell subsets and has been linked to both protective and aggravating effects in disease models. Overall, most studies suggest a pro-inflammatory role for SIRT1 in CD4^+^ T cells, and especially Treg cells, in the setting of autoimmune diseases.

The role of SIRT2, SIRT4, SIRT5, SIRT6, and SIRT7 in CD4^+^ T cells has not been studied to our knowledge. Germline knock-out of SIRT3 resulted in a normal T cell development and response to bacterial and fungal infections in mice (93). Treg cells from FOXP3 cell-specific *Sirt3* knock-out in mice had impaired suppressive function *in vitro* which resulted in an increased cardiac allograft rejection and chronic graft injury *in vivo*. These findings suggest a protective role for SIRT3 in the function of Treg cells (61). Collectively, these data demonstrate that, although opposite effects, both SIRT1 and 3 can be regarded as a potential therapeutic target in autoimmune disease.

## HDAC Inhibition Based Therapy for Chronic Inflammatory Diseases

Acetylation has directly been implicated in the control of cell cycle arrest and apoptosis, making modulators of acetylation such as HATs and HDACs interesting targets for treatment of various diseases, especially cancer. Presently, the therapeutic potential of various HDACi is being tested in clinical trials involving several different malignancies (29, 94–97). For example, the tumor suppressor gene p53, a master coordinator of crucial cellular functions such as apoptosis and genomic stability, is deacetylated by several members of class I and class III HDACs, thereby decreasing its activity (98–102). This underlies the therapeutic potential of HDACi in cancer treatment. HDACi are generally well-tolerated compared to other drugs used in cancer therapy and the most commonly described side effects are gastro-intestinal complaints and fatigue (97, 103). Serious adverse events described include bone marrow depression, liver toxicity, electrolyte disturbances and electrocardiogram (ECG) changes. However, bone marrow depression was shown to be reversible after cessation of the therapeutic agent (97, 104) and intensive monitoring of ECG changes in clinical trials did not show an increase in cardiac adverse events, but long-term follow up is needed (97, 103). Furthermore, of the many HDACi tested in phase I/II clinical trials just a few have been approved for clinical use, which could be caused by absence of selectivity and unclear mechanism of action of many HDACi (97). The broad impact of HDACs on major cell functions, including on CD4^+^ T cell function (both Teff and Treg), suggests that HDACs could be a potential therapeutic target as well for non-malignant diseases like chronic inflammatory diseases. However, especially for non-lethal chronic diseases such as JIA, potential side effects should be carefully studied, monitored and balanced against the possible or expected benefits.

Although on a different scale compared to cancer research, the role of HDACi in the context of autoimmune diseases has also been investigated (Table 1). HDACi were demonstrated to suppress key players of the innate immune system (115–121). For example, Trichostatin A (TSA) and nicotinamide, HDACi inhibiting class I/II or class III HDACs, respectively, decreased the *in vitro* production of IL-6 and TNFα by macrophages from healthy donors and patients with RA after stimulation with TNFα or LPS (115). In mice, oral treatment with the pan-HDACi; suberoylanilide hydroxamic acid (SAHA), also known as Vorinostat, reduced the circulating levels of the pro-inflammatory cytokines TNFα, IL-1β, IL-6, and IFNγ after LPS stimulation (116). Moreover, in a murine lethal LPS-induced septic shock model, treatment with SAHA improved survival by attenuation of several inflammatory markers including neutrophil infiltration in the lungs (117). Furthermore, in LPS stimulated cultured human PBMCs, the class I/II HDACi: ITF2357 (Givinostat) reduced the production of the pro-inflammatory cytokines TNFα, IL-1α, IL-1β, and IFNγ. This *in vitro* data was confirmed in an *in vivo* mouse model where oral treatment with Givinostat reduced LPS-induced serum TNFα and IFNγ by more than 50% (118). Next, HDAC inhibition in RA fibroblast like synoviocytes (FLS) suppressed inflammatory gene expression, including type I IFNγ, IL-6, IL-8 (120, 121) via regulation of cytokine mRNA stability (121). Accordingly, Etinostat (MS275), a class I HDAC inhibitor, selectively affecting HDAC1-3, showed to decrease cell proliferation and secretion of the pro-inflammatory cytokines IL-6 and IL-18 and nitric oxide in cultured human fibroblastic cells from RA patients (119). The nuclear accumulation of NFκ-B was decreased in this model indicating that the anti-inflammatory effect could be mediated via increased acetylation of NFκ-B. A more detailed overview of HDAC inhibition in innate immune cells can be found in literature (51, 52) and is beyond the scope of this review. These data demonstrate that HDAC inhibition of various classes can result in reduced pro-inflammatory cytokine production by innate immune cells, both *in vitro* and *in vivo*.

**Table 1:** Effect of HDAC inhibition on T cell subsets.

**HDAC inhibitor**	**Targeted HDAC member**	**Effect on T-cell subsets**
Trichostatin A (TSA)	Class I and II	- Increase of absolute numbers and percentages of FOXP3^+^ cells in mice with increased FOXP3 acetylation and *Foxp3, Ctla-4* and *Il-10* expression (72). - Increase of FOXP3^+^ cell numbers and percentages in mice. Accompanied with decreased colitis development in mice (73).
Suberoylanilide hydroxamic acid (SAHA)/Vorinostat	Class I, II, and IV	- Increase of FOXP3^+^ cell numbers and percentages and decreased colitis development in mice (73). - Improved cardiac allograft survival in mice, with increased percentage of FOXP3^+^ cells and increased suppressive capacity of these cells. However, different effects with different dosages (105). - Reduced incidence of graft versus host disease in allogenic hematopoietic stem cell transplantation patients in a phase I/II clinical trial. Accompanied by reduced pro-inflammatory cytokines and increased Treg cell numbers and suppressive capacity (106, 107).
ITF2357/Givinostat	Class I and II	- Decreased disease activity and increased survival in SLE prone mice (NZB/W). Increased percentage of Treg cells and decrease in IL-17 producing cells (108). - Decreased joint swelling and cell influx into joint cavity in arthritis mouse model. Reduction in pro-inflammatory cytokines; TNFα, IL-1β (109). - Reduced pro-inflammatory cytokine production after *ex vivo* stimulation of PBMC from healthy volunteers with LPS in a phase I clinical trial (110). - Decreased disease activity scores in 5/9 patients at 12 weeks in a phase II human clinical trial involving children with sJIA (111).
MS275/Etinostat	Class I members HDAC1-3	- No effect on percentage of FOXP3^+^ cells percentage or development of colitis in mice (73).
Valproic acid (VPA)	Class I (primarily) and II	- Increased Treg cell number and suppressive capacity accompanied with a decreased incidence of collagen induced arthritis in mice (112).
Butyrate	Class I, IIa, and IV	- Decreased Th17 cell numbers and increased Treg cell numbers in mice via inhibition of HDAC8. Decreased expression of pro-inflammatory cytokines and reduction in inflammation, bone damage and cartilage damage (113).
EX-527	Class III member; SIRT1	- Increased suppressive capacity of mouse Treg cells *in vivo* (87). - Increased allograft survival and kidney function in a mouse kidney transplant model (114). - Reduction of weight loss and induction of iTreg development in mouse colitis model. Decreased production of IL-17 during *ex vivo* induction of mouse IL-17 cells (92)
Nicotinamide (NAM)/Vitamin B3	Class III member; SIRT1	- Increased percentage of FOXP3^+^cell's *in vitro* in primary human cells (88). - Decreased production of IL-17 during *ex vivo* induction of mouse IL-17 cells (92).

### HDACi Affecting Class I and II HDAC

Next to suppressive effects on innate inflammation, HDACi could be of great interest as therapeutic strategy by suppressing key players of the adaptive immune system. Of special interest for this review is the effect of various HDACi on FOXP3 expression and Treg cell mediated suppression. Treatment of WT mice with the pan-HDACi TSA resulted in an increase of absolute numbers and percentages of CD4^+^FOXP3^+^ cells in lymphoid tissue by increased production in the thymus (72). This was accompanied by an increase in FOXP3 acetylation and expression of Treg associated genes, including *Foxp*3, *Ctla-4*, and *Il-10* (72). In addition, in a mouse colitis model, the pan HDACi TSA and SAHA inhibited the development of colitis, defined by weight loss, diarrhea, bleeding, and histological findings. In line with previous results, this was accompanied by an increase in CD4^+^FOXP3^+^ cells in both absolute numbers and percentages in the lymphoid tissue of these mice (73). Furthermore, in a mouse cardiac transplant model, SAHA treatment prolonged cardiac allograft survival which was associated with an increased percentage of FOXP3^+^ cells in the thymus, lymph nodes and spleen (105). This was combined with an improved suppressive capacity of the Treg cells. However, depending on the dose used, different effects of SAHA on Treg cells were observed. A low dose of SAHA selectively promoted Teff cell apoptosis and hereby increased the relative percentage of Treg cells, while a high dose suppressed the generation of FOXP3^+^ cells (105). Perhaps, this could be explained by differential effects of SAHA concentrations on the various types of HDACs (122). In an arthritis mouse model, Valproic acid (VPA), a strong class I but also class II HDACi, decreased the incidence and disease activity of collagen induced arthritis. In addition, there was an increase in the suppressive capacity and numbers of Treg cells (112). In contrast to TSA and SAHA, Etinostat, a selective inhibitor of HDAC1-3 from class I, had no effect on the development of colitis or on the percentage CD4^+^FOXP3^+^ cells in the colitis mouse model (73). Since TSA is an inhibitor of Class I and II and SAHA of class I, II, and IV HDACs this implies that the inhibition of one or more HDACs from class II are responsible for the increase in Treg cell numbers in this study. As we described above, an important role for HDAC9 in the development of autoimmune disease was demonstrated in several studies. Therefore, HDAC9 inhibition could potentially be responsible for the increased Treg cell numbers and the protective effect in colitis development in these mice treated with TSA and SAHA. HDAC9 inhibition could therefore be of important therapeutic potential in the treatment of autoimmune diseases such as JIA.

In humans, a phase I/II clinical trial aiming to reduce the incidence of graft vs. host disease in patients receiving allogeneic hematopoietic cell transplantation, add-on treatment with Vorinostat (SAHA) reduced pro-inflammatory cytokine levels in plasma and increased Treg cell numbers and suppressive capacity (106, 107).

Although non-specific, a compound known to exert an effect on T cells via HDAC8 inhibition is butyrate (113). Butyrate treatment resulted in a reduction in disease severity in a collagen induced arthritis mouse model. This was associated with decreased expression levels of pro-inflammatory cytokines and a reduction in inflammation scores, bone damage and cartilage damage scores (113). Inhibition of HDAC8 in T cells by butyrate resulted in decreased Th17 cell number and increased number of Treg cells in these mice. However, the effect of butyrate on the immune system seems to be very broad, with the potential to inhibit Class I, IIa, and IV HDACs affecting several cell types, and being not restricted to HDAC inhibition (123–125).

Givinostat, another class I and II HDACi, is together with SAHA one of the few HDACi that has been investigated for its potential therapeutic effect in autoimmune disease in a clinical trial. Both *in vitro* (human primary cells) and in *in vivo* mouse models, Givinostat was demonstrated to have a strong anti-inflammatory effect by affecting key players of the innate and the adaptive immune system (108, 109, 118). The SLE prone NZB/W mice treated with Givinostat showed decreased SLE disease activity and increased survival (108). This was defined by a decrease in anti-nuclear antibodies and immune complex deposition, improvement of renal histopathology, decrease of the pro-inflammatory cytokine IL-1β and increase in the anti-inflammatory cytokine tumor growth factor (TGF)-β. In addition, an increased percentage of Treg cells and a decreased number of IL-17 producing cells was observed in the spleen (108). Furthermore, in an arthritis mouse model, Givinostat treatment showed to decrease joint swelling and cell influx into the joint cavity (109). A reduced production of the pro-inflammatory cytokines TNFα and IL-1β by synovial tissue was demonstrated which resulted in strong inhibition of bone resorption (109). These promising results from pre-clinical (animal) studies resulted in further exploring Givinostat as a potential therapeutic treatment in human autoimmune disease. First, oral treatment with Givinostat was proven to be safe in a phase I trial involving healthy volunteers (110). In addition, the *ex vivo* stimulation of the peripheral blood from these volunteers with LPS showed a reduction in pro-inflammatory cytokine production. Next, in a small phase II trial involving 17 children with systemic onset JIA (sJIA), oral treatment with Givinostat during 12 weeks proved to be relatively safe, with only mild adverse events. Although 4/17 patients discontinued treatment for safety reasons this was reported to be non-drug related. Interestingly, although not set-up and powered to demonstrate efficacy, possible therapeutic effects of treatment were suggested. In the per-protocol treated group, 5/9 patients showed a relevant decrease in disease activity scores (ACRPed50%) at 12 weeks. Moreover, some patients showed a decrease in neutrophil count and a decrease in pro-inflammatory cytokines such as CD40L, IL-1α, and IFNγ in whole blood lysates (111). As this was only a small phase II trial, the therapeutic potential of Givinostat in the treatment of sJIA or other forms of JIA needs to be explored further.

### HDACi Affecting Class III HDAC

The pro-inflammatory role of SIRT1, at least partially caused by an inhibitory effect on FOXP3 stability and function, makes SIRT1 a potentially interesting target for the treatment of autoimmune disease. In line with what was demonstrated in *Sirt1* knock-out mice, selective inhibition of SIRT1 with small-molecule inhibitors promoted Treg cell numbers and function in *in vitro* studies using primary human cells and in *in vivo* animal models for autoimmune diseases (87, 88, 91). Mouse Treg cells treated *in vitro* with the selective SIRT1 inhibitor EX-527 showed a more potent suppressive capacity (87). This was confirmed *in vivo* in a kidney transplant model where mice treated with EX-527 showed increased survival and improved kidney function (114). In addition, in a mouse colitis model, EX-527 treatment resulted in reduced weight loss and promoted the development of induced Treg cells (91). These studies outline the therapeutic potential of SIRT1 inhibition with EX-527 in autoimmune diseases although its safety and efficacy needs to be further investigated in humans.

Even more interesting for potential future use in patients is the relatively specific SIRT1 inhibitor nicotinamide, also known as Vitamin B3 and well-known for many years as a food additive. SIRT1 inhibition via nicotinamide proved to increase percentages of FOXP3^+^ cells *in vitro* in primary human cells (88). Furthermore, both nicotinamide and EX-527 dose dependently decreased the production of IL-17 during *ex vivo* induction of mouse Th17 cells, indicating an important role for SIRT1 in Th17 differentiation (92). Therefore, SIRT1 inhibition, via nicotinamide or EX-527, has the potential to both induce Treg cells and inhibit Th17 cell differentiation, affecting both sides of the disturbed immune balance in autoimmune diseases such as JIA. Importantly, the potential therapeutic effects of nicotinamide in autoimmune diseases is not a new concept, as it has been studied in humans in a variety of diseases for over more than 50 years (126–134). In both adults and children, maintenance therapy with nicotinamide has been associated with improvement of beta-cell function and reduction in pancreatic inflammation in Type 1 diabetes (128, 131, 132, 134, 135). However, results proved variable in other studies and the effects on either Treg or Teff cells in these studies has not been investigated (127, 129, 130, 136). Importantly, in particular for intended future development of nicotinamide maintenance therapy in JIA, the long term use of high dose nicotinamide treatment proved to be safe in multiple clinical trials involving large numbers of adults and children from the age of 5 (130, 132, 136, 137).

## Discussion

In JIA, the distorted immunological balance results in chronic, sometimes lifelong inflammatory arthritis and imposes a significant risk for restricted mobility and even disability in children. Although therapy with DMARDs and biologicals has proven to be very successful in inducing remission in JIA, there is a high percentage of patients that relapse after tapering and stop of maintenance immunosuppressive treatment. Therefore, there is still a medical need for novel treatment strategies that focus on restoring immune tolerance and the prevention of relapses in these children. As increased insight of the mechanisms underlying JIA has revealed that the balance between anti-inflammatory mechanisms such as Treg cells and pro-inflammatory mechanisms such as Teff cells can determine the course of the disease, this balance represents therefore a promising therapeutic target.

HDACs have been demonstrated to directly regulate the differentiation, proliferation and function of CD4^+^ Teff and Treg cells. Therefore, HDACi harbor therapeutic potential to restore immune tolerance and inhibit activation in autoimmune diseases such as JIA. Currently however, literature on HDAC function in T cells and autoimmune disease is mainly focused on mice. HDAC function in humans and autoimmune disease in specific, needs to be further investigated before conclusions about the therapeutic potential of HDACi can be drawn. These mice knock-out studies however help us to better understand the function of specific HDACs in the different T cell subtypes and indicate which HDACs have the best potential as a therapeutic target in autoimmune disease. As HDACs, specifically from class I, have shown to be crucial for the development, differentiation and function of CD4^+^ T cells, the inhibition of some of these HDACs could potentially lead to severe dysregulation of the immune response. This indicates that, in order to use HDACs in the treatment of human autoimmune disease, it is extremely important that HDACi will selectively target specific HDACs. Several pan HDACi, which have a broad effect and target different HDACs and different HDAC classes, have shown a great potential for the treatment of numerous malignancies and pronounced anti-inflammatory effects by affecting both the innate and adaptive immune system both *in vitro* and *in vivo* without affecting general T cell differentiation or development. However, the broad and pronounced anti-inflammatory effect of these pan HDACi certainly hinders the applicability in chronic autoimmune diseases due to the potential side effects. Some of the HDACi however, may show the required selectivity that is needed for treating chronic inflammatory diseases. For example, the class I/II HDACi; Givinostat, was demonstrated to be safe in a small first clinical trial and data from this phase II trial suggested anti-inflammatory effects in sJIA patients, a disease characterized by hyperactivated (innate) inflammatory pathways. In this study, Givinostat seemed to mainly act on effector mechanisms, comparable to the current available treatment options for sJIA. Although promising in this small initial prove of concept trial, the effect of Givinostat treatment in other forms of JIA and the long-term side effects needs to be explored in more extent before it could be considered as part of a potential treatment strategy.

Other HDACi, targeting HDAC6, HDAC9, and SIRT1 not only show suppressive effects on effector mechanisms in inflammation, but may have promising anti-inflammatory effects via improving Treg cell numbers and function as well. The selective and specific inhibition of these HDACs is therefore currently being explored as potential treatment for use in transplant patients suffering GVHD and could be attractive candidates as part of the treatment regimens in chronic autoimmune diseases such as JIA. Although selective deletion of HDAC9 showed promising anti-inflammatory effects, to our knowledge no specific inhibitor of HDAC9 is available yet. In contrast, specific inhibition of HDAC6 is possible and clinical trials involving specific HDAC6 inhibition to prevent allograft rejection in transplant patients are expected in the near future. Since HDAC6i mainly modulate Treg cell numbers and function, the expansion of treatment with specific HDAC6i to other autoimmune diseases seems a logical next step provided that these HDACi have been demonstrated to be safe and in these patients.

Another promising candidate for the treatment of chronic inflammatory diseases such as JIA is the specific inhibition of SIRT1, which showed an anti-inflammatory effect in several models both *in vitro* and *in vivo*. The beneficial effect of SIRT1 inhibition was shown to be the result of induction of Treg cells (numbers) and function as well as inhibition of Th17 function. This means that SIRT1 inhibition positively affects the disturbed immune balance in diseases like JIA in both ways. As nicotinamide is a selective inhibitor of SIRT1, and has already proven to be safe in multiple trials for a range of chronic (inflammatory) diseases, nicotinamide could therefore be considered an attractive compound, even in long term use for human adults and children. This is specifically of interest when used as part of tapering and stop regimens for already proven and effective therapies with either DMARDS and/or biologicals. Long term treatment with both DMARDS and biologicals have potential side effects and can be a burden for both patients (as weekly or bi-weekly injections and for example intolerance complaints for MTX) and society (high costs). Development of novel stop-strategies, directed to decrease the chance of relapse of disease once DMARD and/or biologicals are stopped, by introducing maintenance therapy with nicotinamide as HDACi, are therefore both interesting and attractive in chronic disease such as JIA.

In conclusion, there seems to be potential for HDACi, in particularly for specific HDACi, in restoring immunological tolerance in JIA and other autoimmune diseases. However, the data on HDACi in arthritis and specifically JIA is still very limited and needs to be further explored. When considered (nicotinamide) or proven (other HDACi) safe, these agents could first be potentially used as adjuvant agents in stopping and tapering strategies for conventional immunosuppressive treatment with DMARDS/biologicals. First, the effectivity of HDACi in such strategies need to be tested in double blind randomized controlled clinical trial.

## Author Contributions

LN, JP, SV, and JvL wrote the manuscript. LN, JP, and JvL made figures and table. SV and JvL edited the manuscript.

### Conflict of Interest Statement

The authors declare that the research was conducted in the absence of any commercial or financial relationships that could be construed as a potential conflict of interest.
